# Association between PNPLA3 *rs738409* polymorphism and nonalcoholic fatty liver disease: a systematic review and meta-analysis

**DOI:** 10.1186/s12902-021-00789-4

**Published:** 2021-06-19

**Authors:** Nader Salari, Niloufar Darvishi, Kamran Mansouri, Hooman Ghasemi, Melika Hosseinian-Far, Fateme Darvishi, Masoud Mohammadi

**Affiliations:** 1 Department of Biostatistics, School of Health, Kermanshah University of Medical Sciences, Kermanshah, Iran; 2 Student Research Committee, Kermanshah University of Medical Sciences, Kermanshah, Iran; 3 Medical Biology Research Center, Kermanshah University of Medical Sciences, Kermanshah, Iran; 4 Department of Food Science & Technology, Faculty of Agriculture, Ferdowsi University of Mashhad (FUM), Kermanshah, Iran; 5 Department of Nursing, School of Nursing and Midwifery, Kermanshah University of Medical Sciences, Kermanshah, Iran

**Keywords:** Polymorphism, Gene, Non-alcoholic fatty liver, NAFLD, PNPLA3

## Abstract

**Background:**

Non-alcoholic fatty liver disease (NAFLD) is a common disorder that is known to be the leading cause of chronic liver disease worldwide. This study aims to systematically review and meta-analyze the association between PNPLA3 *rs738409* polymorphism and non-alcoholic fatty liver.

**Methods:**

Following a systematic review and meta-analysis method, articles without any time limitation, were extracted from SID, MagIran, IranDoc, Scopus, Embase, Web of Science (WoS), PubMed and ScienceDirect international databases. Random effects model was used for analysis, and heterogeneity of studies was investigated considering the I^2^ index and using Comprehensive Meta-Analysis software.

**Results:**

The odds ratio of CC genotype in patients with non-alcoholic fatty liver demonstrates the protective effect of CC genotype with the ratio of 0.52, whereas CG genotype presents an increasing effect of CG genotype with the ratio of 0.19, and GG genotype also showed an increasing effect of GG genotype with the ratio of 1.05. Moreover, CG + GG genotypes as a single group demostrated an odds rartio of 0.88.

**Conclusion:**

This meta-analysis highlights that people with CC genotype has 52% lower chance of developing non-alcoholic fatty liver disease, and those with CG genotype had 19% higher risk of developing non-alcoholic fatty liver. Those with GG genotype were 105% more likely to develop non-alcoholic fatty liver than others. Moreover, those present in a population with CG + GG genotypes were 88% more likely to have non-alcoholic fatty liver disease.

## Background

Non-alcoholic Fatty Liver Disease (NAFLD) is a common disorder that is known to be the leading cause of chronic liver disease worldwide. This disorder is caused by abnormal accumulation of fat in liver tissue cells and can eventually lead to liver cirrhosis [1, 2]. The disease was first diagnosed in 1980 and is subsequently being recognised as one of the major contributors to mortality from liver disorders [3].

In people with NAFLD, deaths from liver disease were reported to be 0.77 per thousand people per year, and cardiovascular deaths were 4.79 per thousand people per year [4]. The prevalence of NAFLD in East Asian countries is 15–45% and in the developed Western countries is found to be 20–30% [5]. According to a study in 2010, the prevalence of this disorder in the general population was 35% and in another study in 2019 the prevalence of NAFLD was 25% [6, 7].

The prevalence of NAFLD is associated with disorders such as obesity and insulin-resistant diabetes and metabolic syndrome [4, 8]. In the study of Yamamoto et al., it has been estimated that the number of obese and overweight people will rise to more than 2 billion by the year 2030, so the occurance of NAFLD is expected to increase with the rise in obesity levels in the general population. Moreover, with the prevalence of obesity in children in recent years, NAFLD has been recognised as the most common liver disorder in children [9, 10]. Other causes of non-alcoholic fatty liver disease include sedentary lifestyle, poor diet and genetic polymorphism of different genes [11].

Different types of genes may be involved in the pathogenesis of NAFLD. Genetic factors cause NAFLD in 27 to 39% of cases. One of the most important genetic factors for NAFLD is Single Nucleotide Polymorphism (SNP) (*rs738409*) in the patatin-like phospholipase domain containing protein 3 (PNPLA3). This SNP was first identified in 2008 by two independent studies on the independent genome.

The PNPLA3 *rs738409* C > G SNP is a type of Missense that results in the replacement of cytosine with guanosine and, ultimately, the incorrect coding of methionine rather than isoleucine at position 148. This single nucleotide polymorphism is located in the third exon of the pnpla3 gene. The PNPLA3 gene is located on human chromosome 22 (*chr22q13.31*).

PNPLA3 encodes a protein known as adiponutrin (ADPN). This protein is expressed in adipocytes and hepatocytes. Moreover, this protein has lipolytic and lipogenic properties, however the exact function of adiponutrin is still unclear. PNPLA has also been reported to be highly expressed on human stellate cells. The encoded protein has retinol esterase activity and allows retinol secretion from hepatocytes while the mutation induces intracellular retention of this compound, therefore, PNPLA3 *rs738409* is susceptible to NAFLD.

The function of PNPLA3 *rs738409* is still unknown, however in vitro studies have shown that PNPLA3 protein has tricylglycerol (TG) hydrolase and lysophosphatidyl acyltransferase (LPAAT) and calcium independent phospholipase A2 activities. PNPLA3 also plays a critical role in homestasis of lipid metabolism. PNPLA3 eventually causes glycerolipid hydrolasis in the liver and inhibits lipid outflow into peripheral adipose tissue, thus contributing to hepatic steatosis and related disorders. NAFLD is characterised by the accumulation of lipids in hepatic steatosis.

The PNPLA3 gene is associated not only with liver fat content, but also with hepatic inflammation, hepatic steatohepatitis, fibrosis and cirrhosis, indicating that it plays a key role in the development of NAFLD. Inflammatory infiltration and liver damage are greater in patients carrying PNPLA3 I148M than in wild-type genotype individuals; this gene is thought to be closely linked to liver inflammation. Compared to non-carriers, homozygous carriers has 73% higher liver fat content, 3.2 times higher risk in high necroinflammatory scores and 3.2 times higher risk of developing fibrosis [4, 5, 7, 11–19]. *rs738409* of the patatin-like phospholipase domain containing gene 3 (PNPLA3) is known to be the most common and most potent gene in the development of NAFLD [4].

Furthermore, the association of PNPLA3 gene polymorphisms with other liver disorders such as alcoholic fatty liver (ALD) has also been observed [20]..

Since non-alcoholic fatty liver disease is very common and can have adverse side effects, understanding the factors affecting its occurrence can play a key role in the prevention and control of this disease and the treatment of those affected. This study aims to systematically review and meta-analyse the association between PNPLA3 *rs738409* polymorphism and non-alcoholic fatty liver.

## Methods

### Search method

This study was performed to determine the association between PNPLA3 *rs73409* C > G polymorphism using a systematic review and meta-analysis. Data were collected from Iranian and international databases of Web of Science (WoS), Embase, Scopus, PubMed, science direct, ProQuest, Google Scholar, SID, Irandoc and other international databases. International databses were searched using the keywords (PNPLA3 gene or PNPLA3 polymorphism OR patatin-like phospholipase domain-containing protein3) and (Non-alcoholic Fatty Liver Disease or NAFLD or Nonalcoholic Steatohepatitis) and their possible combination; Persian equivalent of keywords were used for searches within the Persian databases. The Google Scholar search engine was also used with both English and Persian keywords. In order to assess gray literature review, sites related to the subject, as well as the references within the found sources were analysed.

### Criteria for selection and evaluation of articles

Following the search process, all articles were collected in the EndNote software, and all duplicates were removed. Inclusion criteria were: 1- Case control studies, 2- Cohort, and 3- Studies examining the relationship between pnpla3 gene and non-alcoholic fatty liver disease and Exclusion criteria were: 1- Cross-sectional studies, 2- Case reports, 3- Intervention studies, 4- Letters to editor, 5- Studies where the full-text was not available, and 6- Studies in which individuals in the population under study have underlying disease.

Then a list of titles and abstracts was prepared and after hiding the full text of the articles they were provided to the reviewers. Each article was independently reviewed by two reviewers, and in case of disagreement between the two reviewers, the third reviewer’s judgement was considered as the criterion for approval of articles.

During the qualitative evaluation phase, the STROBE checklist was used to evaluate the studies qualitatively. This checklist consists of 22 criteria, of which 18 are used to assess all research papers, and 4 are specific to the type study. The checklist is used to evaluate the study objectives, determination of appropriateness of the sample size, type of study, sampling method, research population, data collection method(s), definition of variables and method of sampling, study data collection tools, study objectives, the statistical test used to assess the findings, and the maximum score derived from this checklist is 32. The articles with a score below 14 were excluded. Studies were then reviewed according to the PRISMA 2009 four-step process, including article identification, screening, eligibility criteria and finally meta-analysis.

### Statistical analysis

In this study, heterogeneity of studies was investigated using I^2^ test, data were analyzed using Comprehensive Meta-analysis software (Biostat, Englewood, NJ, USA version 3), probability of publication bias results were evaluated using both funnel diagrams and Egger test; please note that the significance level was set at 0.05.

## Results

This study investigated the association between PNPLA3 I148M *rs738409* polymorphism and non-alcoholic fatty liver disease through systematic review and meta-analysis. Following searching various databases, a total of 1391 articles entered the study, of which 220 articles were from EMBASE database, 47 articles from ProQuest, 109 articles from PubMed, 84 articles from ScienceDirect, 243 articles from Scopus, 447 articles from Web of Science (WoS), 1 article from SID, 57 articles from Irandoc, 145 articles from Google Scholar, and 2 articles were selected following the reviews of other articles, and were found within the references.

Once the articles were collected, 360 duplicate articles were eliminated, and after reviewing the title and abstracts, 692 other articles were also removed and 339 articles were left subjected to secondary evaluation. After reviewing the full text of the articles in terms of thematic relevance as well as qualitative review of the articles, 308 additional articles were excluded and finally 31 articles entered the meta-analysis process (please see Tables 1 and 2).

**Table 1:** Characteristics of studies entered into the meta-analysis

Row	Author [References]	Publication year	Area	Age of case group	Age of control group	Case Group Size	Control group Size
1	Alam, S [21].	2017	Bangladesh	39.1 ± 8.6	29.64 ± 7.03	99	75
2	Baclig, M. O [22].	2014	Philippines	20–70	20–70	32	36
3	Bhatt, S. P [23].	2013	India	38.2 ± 7	37.1 ± 6.9	162	173
4	Chen, L. Z [24].	2019	China	26.71 ± 2.81	22.48 ± 3.12	512	451
5	Choobini, Neda [19]	2016	Iran	47.9 ± 12.3	40 ± 13.9	95	183
6	Di Costanzo, A [25].	2018	Italy	54	49.7	218	227
7	Gorden, A [26].	2013	America	47 ± 10.6	46 ± 11.8	748	344
8	Hotta, K [27].	2010	Japan	51.7 ± 15	47.2 ± 14.8	253	578
9	Hudert, C. A [28].	2019	Germany	14.11 ± 2.15	46.73 ± 16.3	70	200
10	Karoli, R [29].	2019	India	45 ± 8.2	46 ± 7	100	100
11	Kawaguchi, Takahisa [30]	2012	Japan	52.05 ± 14.85	48.8 ± 16.3	529	932
21	Krishnasamy, N [31].	2020	India	43.15 ± 9.245	41.99 ± 12.7	105	102
13	Lee, S. S [32].	2014	Korea	45.3 ± 15.5	45.3 ± 10.6	155	184
14	Li, Y. L. [33]	2012	China	46.7 ± 13.6	43.1 ± 13.4	203	202
15	Liu, W. Y [34].	2019	China	40.2 ± 12.5	46.6 ± 9.2	349	58
16	Niriella, M. A [35].	2017	Sri Lanka	42–71	(42–71)	1360	391
17	Niu, T. H [36].	2014	China	49.7 ± 16.7	47.69 ± 15.68	390	409
18	Oniki, Kentaro [37]	2015	Argentina	61.2 ± 10.5	67.5 ± 6	393	740
19	Park, J. H [38].	2015	South Korea	48.9 ± 7	49.1 ± 7.2	602	761
20	Peng, X. E [39].	2012	China	45.33 ± 12.48	43.87 ± 13	553	553
21	Rametta, R [40].	2014	Italy	49.7 ± 12.1	47.7 ± 12.1	137	260
22	Shang, X. R [41].	2015	China	11.81 ± 2.20	11.44 ± 2.99	162	865
23	Uygun, A [42].	2017	Turkey	42.1 ± 11.4	34.1 ± 12.8	216	150
24	Valenti, L. [43]	2012	Italy	49.5 ± 12	48(1 ± 2	144	257
25	Valenti, L. [44]	2010	Italy	46.4 ± 11	48.4 ± 13	253	179
26	Vespasiani-Gentilucci, U [45].	2016	Italy	51.5 ± 12.3	40.1 ± 13.1	60	125
27	Wang, C. W [18].	2011	Taiwan	48.11 ± 12.05	45.4 ± 15.93	156	723
28	Wang, X. L. [46]	2016	China	45 ± 13	45 ± 13	376	382
29	Xia, M. F [16].	2016	China	60	61	1385	2915
30	Yang, H. H [47].	2018	China	70.95 ± 4.73	72.53 ± 4.73	97	362
31	Zhang, R. N [48].	2016	China	38.2 ± 13.78	42.64 ± 10.58	59	72

**Table 2:** Overview of CC, CG, GG and CG + GG genotypes based on the obtained studies

Row	Author [References]	Genotype									Dominant		
		CC			CG			GG			CG + GG		
		Case	Control	OR	Case	Control	OR	Case	Control	OR	Case	Control	OR
1	Alam, S [21].	45	37	0.398	27	43	1.365	3	19	5.700	30	62	2.514
2	Baclig, M. O [22].	26	14	0.299	8	12	2.100	2	6	3.923	10	18	3.343
3	Bhatt, S. P [23].	149	112	0.361	16	35	2.704	8	15	2.105	24	50	2.772
4	Chen, L. Z [24].	196	114	0.373	194	236	1.133	61	162	2.959	255	398	2.683
5	Choobini, Neda [19]	15	13	1.776	14	17	2.631	154	65	0.408	168	82	0.563
6	Di Costanzo, A [25].	123	92	0.617	56	91	2.188	48	35	0.713	104	126	1.620
7	Gorden, A [26].	218	411	0.705	103	244	1.133	5	47	4.546	108	291	1.391
8	Hotta, K [27].	175	45	0.498	296	111	0.745	104	97	2.834	400	208	2.057
9	Hudert, C. A [28].	118	20	0.278	71	31	1.444	11	19	6.401	82	50	3.598
10	Karoli, R [29].	51	20	0.240	32	55	2.597	17	25	1.627	59	80	2.780
11	Kawaguchi, Takahisa [30]	247	88	0.553	468	236	0.799	217	203	2.052	685	439	1.759
12	Krishnasamy, N [31].	59	19	0.261	29	50	2.288	14	36	3.280	43	86	6.211
13	Lee, S. S [32].	55	31	0.586	92	75	0.938	37	49	1.837	129	124	1.705
14	Li, Y. L. [33]	94	70	0.605	90	84	0.878	18	49	3.253	108	133	1.654
15	Liu, W. Y [34].	24	94	0.522	24	152	1.093	8	85	2.012	32	237	1.719
16	Niriella, M. A [35].	25	54	0.605	134	464	0.993	232	842	1.114	366	1306	1.652
17	Niu, T. H [36].	183	48	0.173	176	153	0.855	50	189	6.751	226	342	5.769
18	Oniki, Kentaro [37]	223	38	0.248	394	111	0.346	121	45	0.662	515	156	0.288
19	Park, J. H [38].	280	172	0.678	364	293	1.034	117	137	1.622	481	430	1.455
20	Peng, X. E [39].	235	183	0.669	259	276	1.131	59	93	1.693	318	369	1.482
21	Rametta, R [40].	150	51	0.435	95	67	1.662	15	19	2.630	110	86	2.299
22	Shang, X. R [41].	338	60	0.917	418	74	0.899	109	28	1.449	527	102	1.090
23	Uygun, A [42].	85	64	0.322	50	90	1.429	15	62	3.623	65	152	3.106
24	Valenti, L.(2012) [43]	146	55	0.470	95	68	1.526	16	21	2.572	111	89	2.128
25	Valenti, L.(2010) [44]	118	103	0.355	56	114	1.801	5	36	5.773	61	150	2.817
26	Vespasiani-Gentilucci, U [45].	83	29	0.473	34	18	1.147	8	13	4.045	42	31	2.112
27	Wang, C. W [18].	269	40	0.582	335	80	1.219	119	36	1.523	454	116	1.718
28	Wang, X. L. [46]	169	122	0.605	174	191	1.234	39	63	1.770	213	254	1.652
29	Xia, M. F [16].	1200	486	0.773	1363	684	1.111	352	215	1.338	1715	899	1.294
30	Yang, H. H [47].	110	27	0.884	123	40	1.364	129	30	0.809	252	70	1.132
31	Zhang, R. N [48].	32	12	0.319	31	27	1.116	9	20	3.590	40	47	3.133

**Fig. 1:**
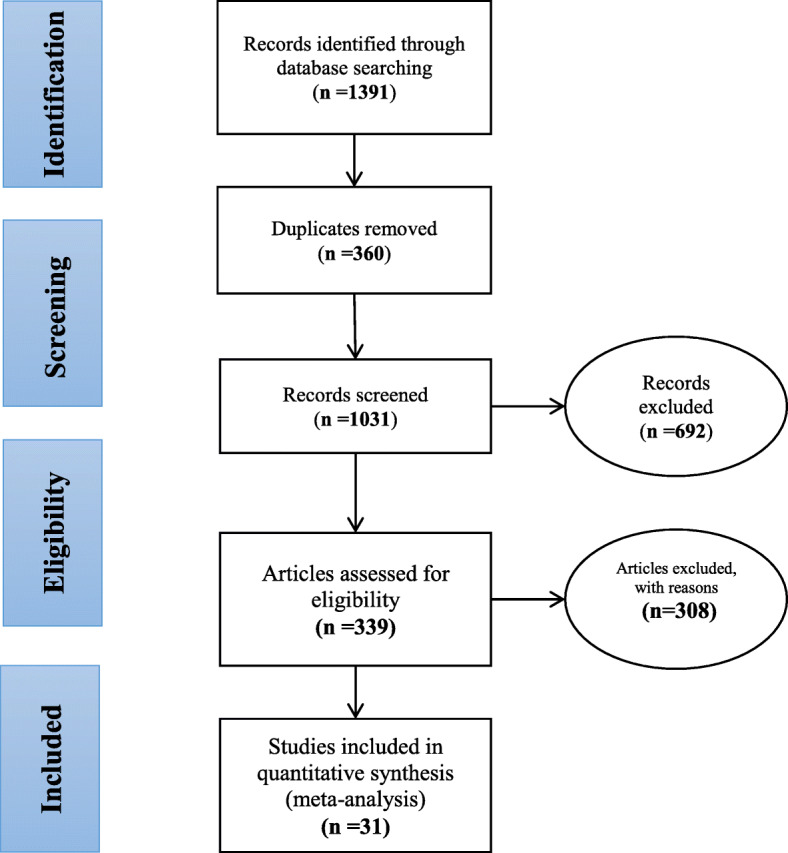
PRISMA flow diagram for the processes followed for includign studies in this systematic review and meta-analysis

The PRISMA 4-step process highlighting the processes in obtaining the final articles for our meta-analysis is presented in Fig. 1.

### Investigation of heterogeneity and publication bias (CC genotype)

**Fig. 2:**
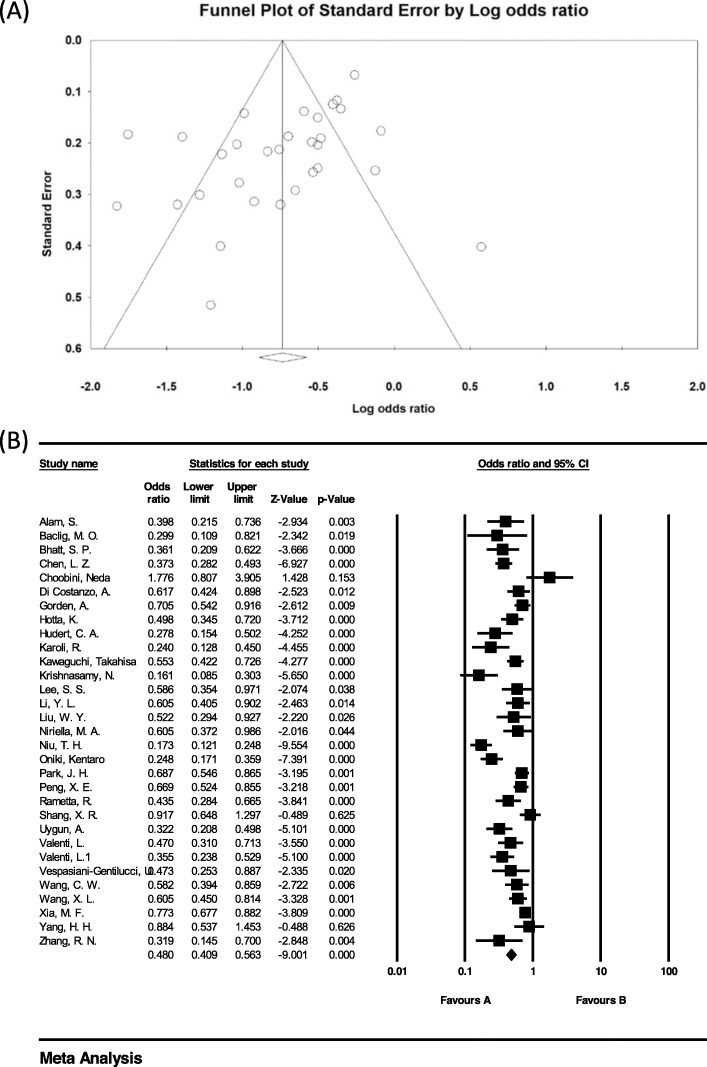
Funnel plot (**A**) and Overall forest plot of CC Genotype in Patients with Non-Alcoholic Fatty Liver Based on Random Model (**B**)

The heterogeneity of the studies was evaluated using the I^2^ test. Based on this test, I^2^ = 82.2% was obtained, which indicates high heterogeneity in the included studies. Moreover, the results of the publication bias study were compared with the Egger test (please see Fig. 2 A), which was not statistically significant (*P* = 0.052).

The total number of samples included in the case group and in the control group were 9973 and 13,048 respectively. The odds ratio of CC genotype in patients with non-alcoholic fatty liver was 0.48 based on meta-analysis (95% CI: 0.40–056), indicating an protective effect of CC genotype with 0.52, meaning that those with this genotype are 52% less likely to develop non-alcoholic fatty liver than others. In Fig. 2 B, the odds ratio based on the random effects model is shown where the black small rectanlges has the odds ratio and the rectangle length indicates the 95% confidence interval; the diamond shape represents the odds ratio for the entire study (Fig. 2 B).

### Investigation of heterogeneity and publication bias (CG genotype)

**Fig. 3:**
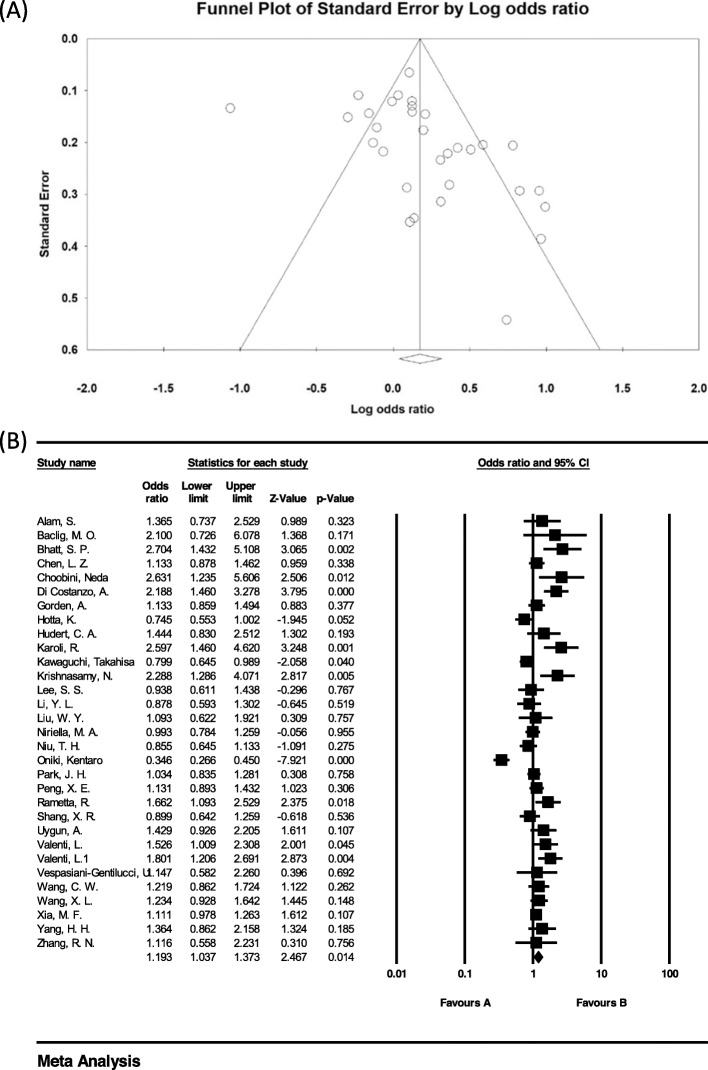
Funnel plot (**A**) and Overall forest plot of CG Genotype in Patients with Non-Alcoholic Fatty Liver Based on Random Model (**B**)

The heterogeneity of the studies was evaluated using the I^2^ test. Based on this test, I^2^ = 80.3% was obtained, which indicates high heterogeneity in the included studies. Moreover, the results of the publication bias study were compared with the Egger test (please see Fig. 3 A), which was not statistically significant (*P* = 0.072).

The total number of samples included in the case group and in the control group were 9973 and 13,048 respectively. The odds ratio of CG genotype in patients with non-alcoholic fatty liver was 1.19 based on meta-analysis (95% CI: 1–1.33), indicating an increasing effect of CG genotype with 0.19, meaning that those with this genotype are 19% more likely to develop non-alcoholic fatty liver than others. In Fig. 3 B, the odds ratio based on the random effects model is shown where the black small rectanlges has the odds ratio and the rectangle length indicates the 95% confidence interval; the diamond shape represents the odds ratio for the entire study (Fig. 3 B).

### Investigation of heterogeneity and publication bias (GG genotype)

**Fig. 4:**
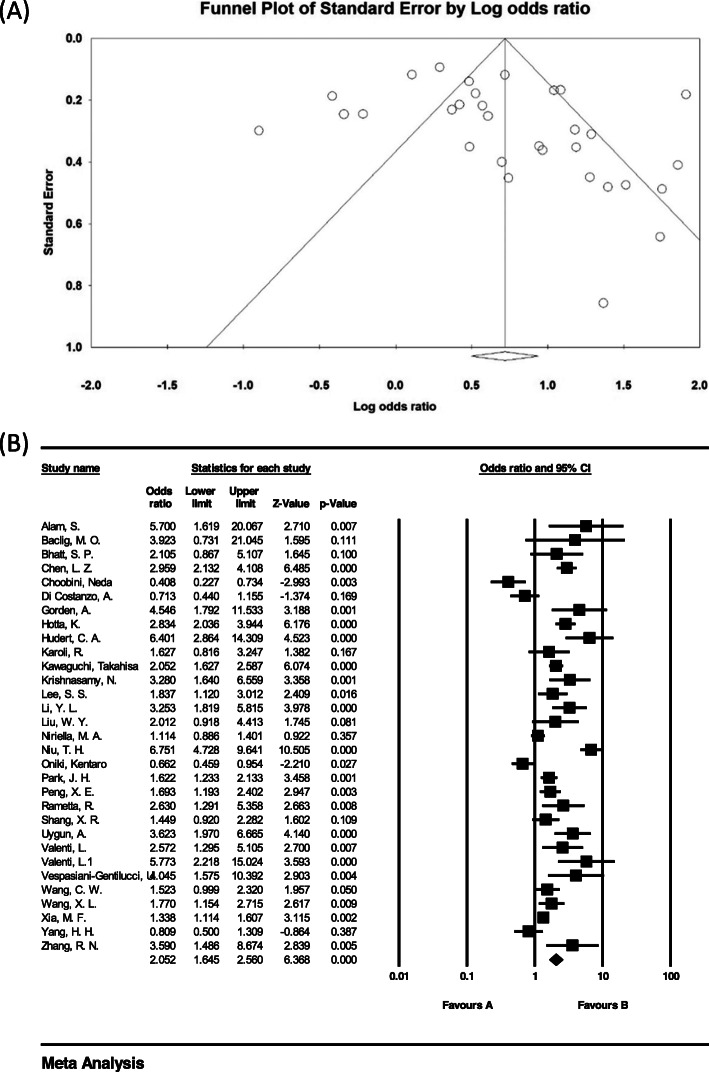
Funnel plot (**A**) and Overall forest plot of GG Genotype in Patients with Non-Alcoholic Fatty Liver Based on Random Model (**B**)

The heterogeneity of the studies was evaluated using the I^2^ test. Based on this test, I^2^ = 86.3% was obtained, which indicates heterogeneity in the included studies. Moreover, the results of the publication bias study were compared with the Egger test (please see Fig. 4 A), which was not statistically significant (*P* = 0.064).

The total number of samples included in the case group and in the control group were 9973 and 13,048 respectively. The odds ratio of GG genotype in patients with non-alcoholic fatty liver was 2.05 based on meta-analysis (95% CI: 1.64–2.56), indicating an increasing effect of GG genotype with 1.05, meaning that those with this genotype are 105% more likely to develop non-alcoholic fatty liver than others. In Fig. 4 B, the odds ratio based on the random effects model is shown where the black small rectanlges has the odds ratio and the rectangle length indicates the 95% confidence interval; the diamond shape represents the odds ratio for the entire study (Fig. 4 B).

### Investigation of heterogeneity and publication bias (CG + GG genotype)

**Fig. 5:**
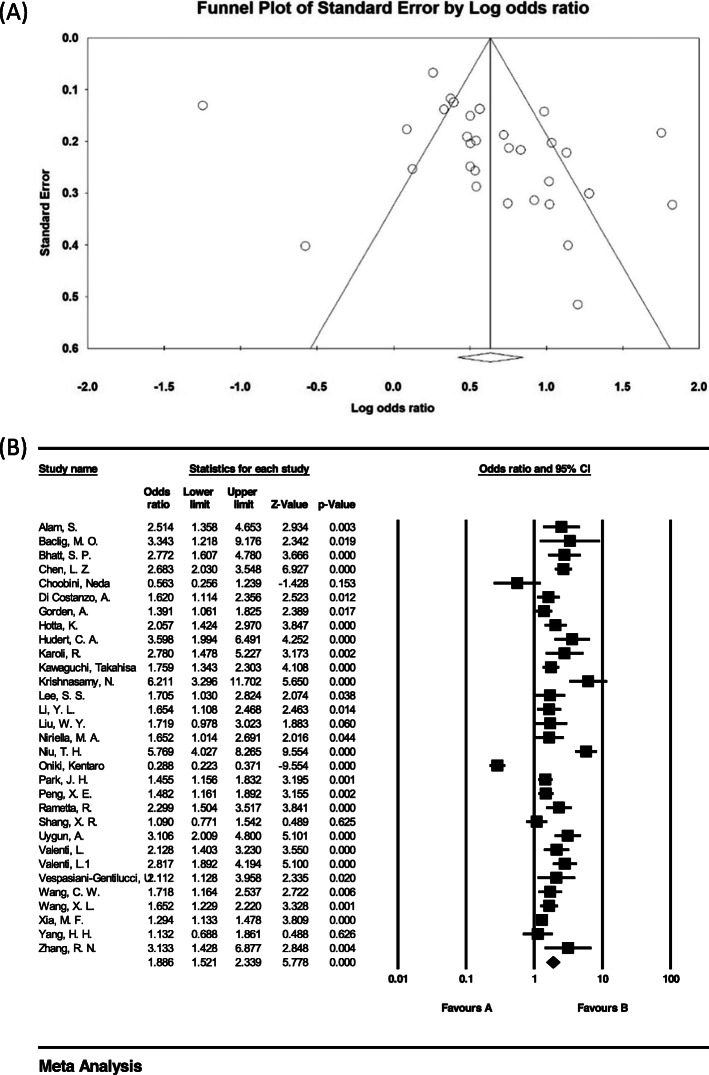
Funnel plot (**A**) and Overall forest plot of CG + GG Genotype in Patients with Non-Alcoholic Fatty Liver Based on Random Model (**B**)

The heterogeneity of the studies was evaluated using the I^2^ test. Based on this test, I^2^ = 90.7% was obtained, which indicates heterogeneity in the included studies. Moreover, the results of the publication bias study were compared with the Egger test (please see Fig. 5 A), which was not statistically significant (*P* = 0.054).

The total number of samples included in the case group and in the control group were 9973 and 13,048 respectively. The odds ratio of CG + GG genotype in patients with non-alcoholic fatty liver was 1.88 based on meta-analysis (95% CI: 1.5–2.3), indicating an increasing effect of CG + GG genotype with 0.88, meaning that those with this genotype are 88% more likely to develop non-alcoholic fatty liver than others. In Fig. 5 B, the odds ratio based on the random effects model is shown where the black small rectanlges has the odds ratio and the rectangle length indicates the 95% confidence interval; the diamond shape represents the odds ratio for the entire study (Fig. 5 B).

## Discussion

In this study, after investigating the association between different genotypes of PNPLA3 *rs738409* polymorphism and non-alcoholic fatty liver disease, we highlighted that people with CC genotype with the odds ratio of 0.48, have 52% lower risk of developing non-alcoholic fatty liver, while this ratio in CG and GG genotypes were 1.19 and 2.05 respectively, and therefore the probability of developing the disease in those with these genotypes were 19% (CG) and 105% (GG) higher. On the other hand, considering the CG + GG groups as a single population/group, and following a statistical analysis, it was concluded that the odds ratio of this group in relation to occurance of Non-alcoholic fatty liver was 1.88, meaning that this group were 88% more likely to develop the disorder than others. The effect of the G allele on non-alcoholic fatty liver disease can also be emphasized. A study in India in 2020 also found that the G allele plays a key role in the development of NAFLD [31].

NAFLD is recognised as one of the most common liver diseases in the world with unknown etiology and pathogenesis. However, several factors including genetics, diet and inactivity, have been presented as some of the key reasons for the development of the disease. It has also been found that a good diet and regular exercise can reduce the risk of developing insulin resistance and can boost glucose homeostasis. Other SNPs such as rs2896019 and rs3810322 have also been reported to increase the risk of non-alcoholic fatty liver disease [1]. Past genomic studies have identified two genes *PNPLA3 I148M* and *TM6SF2 E167K* as the most likely genetic factors in the development of NAFLD [49].

According to meta-analysis by Zhang et al. (2015) on some studies undertaken in Asian countries, when comparing people having the G allele with a population with the C allele, the probability of non-alcoholic fatty liver disease was reported to be 1.92, and therefore it was concluded that the G allele is likely to increase the development of non-alcoholic fatty liver to the liver in people with G allele by 92%; Moreover, it can increase the risk of renal fibrosis and ALT serum levels. Development of NAFLD in the dominant phenotype (CG + GG) was 110% higher than the recessive phenotype. On the other hand, comparing the CG + GG populations with the CG genotype, it was concluded that the risk of NAFLD was higher in the homozygous GG population than in other populations [5].

Another meta-analysis conducted in 2019 stated that this polymorphism had a major impact on the development of tissue damage in liver and that the G allele was considered as a risk factor for NAFLD in such a way that the ratio of development of the disease in those with one G allele to those without it was 1.88, and 4.01 in those where both alleles were G. It has also been suggested that this gene increases alanine aminotransferase levels in serum [50].

According to a meta-analysis by Jiaying et al. (2020), this gene is involved in the development of non-alcoholic osteopathy (NASH) in children and adolescents; it is also accosiated with factors such as serum alanine transaminase, aspartate transaminase, gamma glutamyl transferase, that are indicators of liver damage [51].

Another meta-analysis in 2015, it was reported that all genetic variations in the *rs738409* polymorphism in the pnpla3 gene was strongly associated with the incidence of NAFLD and NASH, especially in Asian and Spanish populations. In this study, however, no association was found between *rs738409* polymorphism and hepatic steatosis. It was also reported that the GG genotype had a high impact on the development of NAFLD as well as renal fibrosis. The ratio of this genotype over inflammation occurance was reported as 3.13 [14].

According to a study by Chobin et al., the CG genotype was identified as a predisposing genotype to a 2.63-fold increase in the likelihood of developing the disease. Moreover, it was reported that the GG genotypes possess a protective effect, meaning that existence of such gentype results in a 59% decrease in developing NAFLD. Furtheremore, the odds of developing the disease in the CC genotype was 1.78 [19].

According to another study by Sood et al. (2016) in Japan, the odds ratio of the GG genotype was 36.5% in obese people and 47.8% in the non-obese population who had a fatty liver. Moreover, the modified odds ratio of non-alcoholic fatty liver disease in GG genotype was reported to be 4.15 in non-obese individuals, and 2.76 in obese pupulation. This genotype also increases the chances of developing steatosis and liver fibrosis [52]. A family history of NAFLD may result in higher levels of ALT and cholesterol among children. Moreover, it was reported that for every 10 unit increase in ALT (in IU / L) there will be approximately 1.5 times and for every 20 unit (mg / L) increase in body cholesterol, there will be approximately 2 times the risk of developing NAFLD in children [53, 54].

### Limitation

The limitation of this study was the lack of access to the full-text of some of the sources.

## Conclusion

This meta-analysis study demonstrated that people with the CC genotype were 52% less likely to develop non-alcoholic fatty liver disease, and people with CG genotype were 19% more likely to develop non-alcoholic fatty liver. Moreover, population with the GG genotype, had 105% more chance of developing a non-alcoholic fatty liver. Moreover, population with CG + GG genetypes demonstrate 88% more chance of developing the disease, and this is suggesting the effect of G allele on non-alcoholic fatty liver disease. In future, the effects of genetic and environmental factors on the level fo tissue damage, and also the effect of this gene on fibrosis and liver cirrhosis can be studied.

## Data Availability

Datasets are available through the corresponding author upon reasonable request.

## Abbreviations

NAFLD: Non-alcoholic fatty liver disease
SNP: Single Nucleotide Polymorphism
TG: Tricylglycerol
WoS: Web of Science
PRISMA: Preferred Reporting Items for Systematic Reviews and Meta-Analysis.
STROBE: Strengthening the Reporting of Observational Studies in Epidemiology for cross- sectional Study
